# Assessment of the contribution of morbidity and mortality conferences to quality and safety improvement: a survey of participants’ perceptions

**DOI:** 10.1186/s12913-016-1431-5

**Published:** 2016-05-11

**Authors:** André Lecoanet, Gwenaëlle Vidal-Trecan, Frédéric Prate, Jean-François Quaranta, Elodie Sellier, Alizé Guyomard, Arnaud Seigneurin, Patrice François

**Affiliations:** Public Health Department, University Hospital, Grenoble, F-38043 France; Public health unit: Risk Management and Quality of Care, Paris Centre University Hospital Group, AP-HP, Paris, F-75014 France; Research Unit: METHODS team, INSERM U1153 (Centre de Recherche Epidémiologie Biostatistique, Sorbonne Paris Cité), Paris, F-75005 France; Medical School, Paris Descartes University, Paris, F-75006 France; Public Health Department, University Hospital, Nice, F-06003 France; Research Unit TIMC-IMAG (UMR 5525 CNRS/UJF-Grenoble 1), Grenoble, F-38041 France

**Keywords:** Morbidity and mortality conferences, Patient safety, Quality improvement, Hospital

## Abstract

**Background:**

Evidence for the effectiveness of the morbidity and mortality conferences in improving patient safety is lacking. The aim of this survey was to assess the opinion of participants concerning the benefits and the functioning of morbidity and mortality conferences, according to their organizational characteristics.

**Methods:**

We conducted a survey of professionals involved in a morbidity and mortality conference using a self-administered questionnaire in three French teaching hospitals in 2012. The questionnaire focused on the functioning of morbidity and mortality conferences, the perceived benefits, the motivations of participants, and how morbidity and mortality conferences could be improved. The perception of participants was analysed according to the characteristics of morbidity and mortality conferences.

**Results:**

A total of 698 participants in 54 morbidity and mortality conferences completed the questionnaire. Most of them (91 %) were satisfied with how the morbidity and mortality conference they attended was conducted. The improvements in healthcare quality and patient safety were the main benefits perceived by participants. Effectiveness in improving safety was mainly perceived when cases were thoroughly analysed (adjusted odds ratio [a0R] =2.31 [1.14–4.66]). The existence of a written charter (*p* = 0.05), the use of a standardized case presentation (*p* = 0.049), and prior dissemination of the meeting agenda (*p* = 0.02) were also associated with the perception of morbidity and mortality conference effectiveness. The development and achievement of improvement initiatives were associated with morbidity and mortality conferences perceived as being more effective (*p* < 0.01). Participants suggested improving the attendance of medical and paramedical professionals to enhance the effectiveness of morbidity and mortality conferences.

**Conclusions:**

Morbidity and mortality conferences were positively perceived. These results suggest that a structured framework and thoroughly analyzing cases improve their effectiveness.

## Background

Morbidity and mortality conferences (MMCs) were established in the United States at the beginning of the 20th century and have become a relevant part of physician education [1]. They were initially set up by surgeons and anaesthesiologists and became a way to fulfill the mandatory requirement for assessment of physicians’ practices. With the emergence of the management of patient safety in the healthcare system in the 1990s, several authors suggested that MMCs could be conducted to improve the quality and safety of healthcare [2]. In this context, MMCs were adopted in France. In 2010, the French health authorities decided to make MMCs mandatory in high-risk specialties such as surgery, anaesthesia, intensive care and oncology. Fulfilling this commitment was a key factor in the accreditation of hospitals.

Therefore, MMCs are now widely implemented in hospitals, but evidence of their effectiveness in improving patient safety is lacking. Numerous authors have found that the characteristics of MMCs varied greatly [3–5] with variations in their goals, their structures, and their processes.

The best evidence of the effectiveness of MMCs would be to demonstrate an effect on a patient-related outcome. However, this approach presents methodological issues and very few studies have concluded in a significant effect of MMCs on a clinical outcome [6–8]. Considering the wide variety of situations, choosing and using a patient-related outcome is difficult. Where this is possible, the low incidence of specific events would lead to a lack of statistical power [9]. In this context, several authors estimated the effectiveness of MMCs indirectly through the perception of the participants in these meetings [4, 10, 11]. They assessed their functioning and their impact in terms of knowledge or improvement of practices. Some authors emphasized the wide variety of leadership structures and the perceived effectiveness [4]. They suggested that a formalized framework should lead to a better perception of effectiveness and more satisfying MMCs [4, 10, 11].

The present survey focused on the functioning of MMCs, the perception of their benefits by the participants, especially concerning healthcare quality and safety improvement, the personal motivations of participants, and suggestions for improvement. The main objective was to assess the participants' opinion concerning the effectiveness of MMCs as a tool to improve patient safety. Within a secondary objective, we identified the characteristics of MMC functioning associated with the perception that the MMC produced benefits.

## Methods

### Study design

This observational study was conducted in three teaching hospitals in France: Grenoble, Nice, and Cochin (within the Paris Hospital network) in 2012 and was based on self-administered questionnaires.

### Population

The MMCs, established for more than 1 year, were identified between September and December 2010 in each hospital. Every professional involved in one of these MMCs who agreed to participate was included. The protocol of the study was first described to all MMC leaders. It was then presented and discussed within each MMC and the team agreed to the collection of information from all participants to assess their opinion on the MMC. According to the French law, no ethics review was necessary for this study considering that there was no intervention or collection of personal data [12].

### Data collection

The questionnaire was based on the main items used by Aboumatar *et al.* to interview the MMC leaders at the John Hopkins Hospital [4]. A total of 26 multiple-choice questions explored the perception of the benefits of the MMC, the functioning of the MMC, personal motivations, and possible improvements. Response choices followed a four-modality Likert scale from "entirely disagree" to "entirely agree". A free comment field ended the questionnaires.

The questionnaire was provided to any professional who attended at least one MMC meeting. They were delivered electronically by email or by hand at the end of a meeting. In case of electronic delivery, a reminder was sent if no reply was recorded within 15 days.

The characteristics of MMCs were collected in a previous study [13] by analyzing all the documents produced by MMCs (i.e., charter or organizational procedure, meeting reports, annual activity report, and documents related to implemented improvement initiatives) and through the observation of one meeting per MMC. The main characteristics collected were related to formalization (the presence of a written charter, an annual activity report, a list of participants and a meeting schedule, the exhaustiveness of the meeting minutes, the prior dissemination of the meeting agenda, the standardized case presentation using slides), the analysis of cases (the thoroughness of the analysis of the failure, the number of cases presented, the length of the meetings, the practice of thematic meetings), the attendance rate of occupational groups and generalities (time the MMC had existed, department specialty). The thoroughness of the analysis of the failure was coded as "0 - failures were not examined, 1- failures were examined, 2 - failures were thoroughly analysed searching for causes". Other categorical characteristics were considered as present or absent.

Furthermore, each improvement initiative decided during the study period was followed for 1 year. The implementation of initiatives and the impact were assessed on the basis of MMC leaders’ interviews and document analysis. Assessment was carried out using a score from 1 to 6, according to the designation of a person in charge (0-1), the definition of a timeline (0-1), the completion of each initiative (0-1-2) and its evaluation (0-1-2). These scores were aggregated to obtain an effectiveness index for each MMC.

### Data analysis

Descriptive statistics were performed using numbers and percentages. Ordinal and continuous variables, especially the effectiveness index and the number of improvement initiatives, were dichotomized according to their median.

We compared the proportion of agreement according to the main characteristics of MMC operation, the number of improvement initiatives and the effectiveness index. With an expected high rate of “at least agree” responses, we defined the agreement level as “totally agree” in order to avoid a ceiling effect.

Univariate analysis was performed using Fisher exact tests. A multivariable analysis was carried out to identify the independent characteristics associated with the perception of patient safety improvements. A mixed effect logistic regression was used to take into account the multilevel structure of the data with individuals attending MMCs. The characteristics associated with outcomes in univariate analysis with a *p*-value less than 0.2 were entered in the model. The number of actions undertaken and the effectiveness index were excluded because they reflected the effectiveness of the MMC rather than an operational characteristic. Two-sided *p*-values of less than 0.05 were considered statistically significant. Analyses were performed using STATA 11.0 software.

The free comments were analysed by two independent investigators. The content of each comment was first classified according to three categories: positive comments, negative comments, and suggestions. Secondly, each part of the comments was organized by theme. Differences in categories and themes were discussed until a consensus was reached.

## Results

We identified 1134 professionals involved in 54 MMC groups, including 24 MMCs in medical units, 20 in surgical units, and 10 in anaesthesiology/intensive care units. Median time MMCs had existed was 4 years (IQR, 3–5). A total of 698 (61.6 %) participants completed the questionnaire: 395 (58 %) senior practitioners, 158 (23.2 %) residents, 60 (8.8 %) head nurses, 28 (4.1 %) nurses, 24 (3.5 %) midwives, and 16 other paramedical or administrative professionals. The type of occupation was missing for 17 respondents. Two hundred eighty (40.1 %) worked in a medical unit, 219 (31.9 %) in a surgical unit, and 199 (28.5 %) in an anaesthesiology/intensive care unit. Response rates were similar for the different specialties (62.5 % in medicine, 62.5 % in surgery, 59.1 % in anaesthesiology/intensive care, *p* = 0.55).

Most of the respondents agreed totally or partially that the meeting was managed satisfactorily (89.9 %, *n* = 617) and was non-blaming (86.1 %, *n* = 589). More than 80 % of the respondents agreed totally or partially for each benefit proposed, except improvement of the relationship between medical and paramedical teams (60.6 %, *n* = 421).

Considering only respondents who totally agreed, the perceived benefits were mainly quality of care improvement and patient safety improvement (Table 1). Personal motivations to participate in MMCs were also most often related to improvement of individual and team practices. Only a minority of participants totally agreed with the suggested areas for improvement. The highest proportion of agreement concerned the increase of paramedical professionals’ participation. There were significant differences in opinion of the various occupational groups, especially for the perceived benefits. Senior physicians and paramedical professionals had a better opinion of the benefits than did residents. Personal motivations also differed with senior physicians more often motivated by improvements in practices than residents. Senior physicians more frequently agreed that MMCs were associated with an educational aspect for initial and continuing education. They more often suggested improving the case analysis method. Paramedical staff more often acknowledged the benefits in terms of practices and improvements of organizational and relational aspects. Paramedical personnel also more often agreed with increasing the participation of the paramedical staff.

**Table 1:** Proportion of agreement for each question according to the professional status of MMC participants

	Total *N* = 698^a^ (%)	Senior physicians *N* = 395 (%)	Residents *N* = 158 (%)	Others^b^ *N* = 128 (%)	*p*
Perceived benefits					
Initial education	37.0	42.3	30.4	28.6	0.004
Continuing education	42.4	50.0	31.0	35.1	<0.001
Improvement of quality of care	61.9	66.8	51.3	67.2	0.002
Improvement of patient safety	63.2	67.8	52.5	68.7	0.002
Standardization of medical practices	45.2	47.8	27.8	60.8	<0.001
Application of clinical guidelines	37.2	37.9	26.3	52.1	<0.001
Improvement of functioning in the unit	47.7	50.8	35.0	55.1	0.001
Improvement of teamwork	33.0	36.5	17.7	43.8	<0.001
Improvement of relations between medical and paramedical teams	22.8	21.9	12.7	42.5	<0.001
Improvement of safety culture	35.9	39.4	22.1	44.7	<0.001
Discussion of collective errors	54.8	55.2	44.3	67.2	0.001
Functioning assessment					
Friendly environment	40.1	44.8	29.7	38.2	0.004
Avoidance of blame	35.9	38.4	29.9	33.3	0.15
Satisfactory conduct	34.1	40.4	24.4	37.6	0.002
Personal motivations					
Fulfillment of mandatory training requirement	12.2	12.7	12.3	9.9	0.78
Social interactions	15.7	17.1	11.5	18.8	0.17
Improve professional practices	71.6	75.9	58.0	69.0	<0.001
Improve team practices	75.6	82.2	56.3	74.6	<0.001
improve of functioning in the unit	66.1	72.3	50.6	68.5	<0.001
Areas for improvement					
Increasing the number of meetings	6.7	6.2	7.6	4.2	0.50
Increasing the participation of senior physicians	22.6	24.0	15.4	22.0	0.10
Increasing the participation of the unit head	16.2	18.1	10.8	17.8	0.10
Increasing the participation of invited specialists	26.4	29.5	22.9	21.0	0.10
Increasing the participation of paramedical professionals	31.9	31.0	21.5	42.6	0.001
Improving the case selection method	16.4	17.1	12.8	12.9	0.36
Improving the case analysis method	14.6	17.2	7.6	12.1	0.01

^a^ Includes 17 participants whose occupation was missing

^b^ Includes midwives, nurses, head nurses, and other paramedical professionals

Agreement is defined as "totally agree" response

There were also differences in the perceived benefits of MMCs, depending on the specialty of the department. MMCs in medical specialties had a greater perceived impact on the relationship between medical and paramedical teams (29.5 % versus 20.9 %, *p* = 0.03). Surgical MMCs had a greater perceived impact on initial education (45.8 % versus 33.3 %, *p* < 0.01). Participants in MMCs in anaesthesia/intensive care units were less likely to consider their MMC as non-blaming (27.7 % versus 38.2 %, *p* = 0.03).

Considering how long MMCs had existed, there were no differences in terms of perceived benefits and motivations. However, participants in the oldest MMCs were less likely to consider their MMC as non-blaming (50.2 % versus 59.1 %, *p* = 0.03).

The characteristics of MMCs significantly associated with a better opinion were the prior dissemination of the meeting agenda, the existence of a written charter, the use of a standardized case presentation, the examination of failures during the discussion, and fewer cases presented (Table 2). In addition, a better opinion was significantly associated with a higher effectiveness index based on planning, implementation, and assessment of improvement initiatives.

**Table 2:** Proportion of agreement with the perception of patient safety improvement, according to MMC operational characteristics

	MMCs contribute to improving patient safety		
	If characteristic is absent *N* ^a^ (%)	If characteristic is present *N* ^a^ (%)	*p*
Formalization			
Written charter	﻿38 (53.6)	412 (65.8)	0.049
Yearly activity report	76 (59.8)	374 (65.6)	0.22
List of participants	76 (63.9)	374 (64.7)	0.92
Exhaustiveness of meeting minutes	57 (62.64)	393 (64.9)	0.73
Prior dissemination of meeting agenda	156 (59.1)	291 (68.0)	0.02
Established meeting schedule	248 (65.4)	202 (63.5)	0.63
Standardized presentation using slides	153 (57.9)	297 (68.6)	0.005
Content, analysis			
Failures are			
examined	24 (42.1)	426 (66.6)	<0.001
and thoroughly analysed	292 (61.3)	158 (71.5)	0.01
Few cases presented (<19)	206 (59.2)	244 (69.9)	0.03
Longer meetings (>80 min)	234 (63.9)	216 (65.3)	0.75
Thematic meetings organized	359 (63.0)	91 (71.6)	0.07
Attendance rates (if greater than the median)			
Senior physicians	226 (64.2)	224 (64.9)	0.87
Head nurses	255 (63.6)	195 (65.9)	0.57
Nurses	195 (62.1)	255 (66.6)	0.23
Older group (≥4 years of experience)	198 (65.13)	252 (64.12)	0.81
Improvement initiatives			
Effectiveness index (≥10)^b^	185 (59.5)	265 (68.6)	0.01
Number of initiatives (>2)	144 (60.8)	306 (66.5)	0.13

^a^Count of "totally agree" responses

^b^The effectiveness index was a composite score based on planning, implementation, and assessment of improvement initiatives

A greater perception of benefits in terms of continuous training was associated with the use of standardized case presentations (48.4 % versus 34.3 %, *p* < 0.01), a higher effectiveness index (48.2 % versus 36.8 % %, *p* < 0.01), and more decisions on initiatives (47.2 % versus 35.2 %, *p* < 0.01).

Participants in MMCs examining failures, without analysis, were significantly less likely to consider their MMC as non-blaming compared to those participating in MMCs that did not examine failures (34.0 % versus 49.1 %, *p* = 0.03). However, this difference tended to disappear when the failures were accurately analysed (36.7 % versus 34.54 %, p = 0.60).

Participants from MMCs with a greater effectiveness index more frequently perceived benefits in terms of continuing education (48.2 % versus 36.8 %, *p* < 0.01), standardization of practices (51.3 % versus 39.4 %, *p* < 0.01), and application of guidelines (41.6 % versus 33.3 %, *p* < 0.01).

In multivariable analysis, a thorough analysis of the causes of failures was independently associated with a greater perception of improvement of patient safety (Table 3).

**Table 3:** Multivariable analysis of safety improvement perception according to MMC operational characteristics

	Adjusted OR [95 % CI]	*p*
Written charter	1.30 [0.72–2.36]	0.38
Prior dissemination of meeting agenda	1.10 [0.74–1.62]	0.63
More cases presented	0.76 [0.53–1.10]	0.15
Thematic MMC	1.25 [0.76–2.08]	0.38
Standardized presentation	1.14 [0.77–1.68]	0.51
Failures are		
examined	1.85 [0.96–3.55]	0.06
and thoroughly analysed	2.46 [1.20–5.03]	0.01

A total of 110 respondents (15.8 %) wrote a comment after having answered the structured questionnaire. Fourteen highlighted the value of MMCs and three others underscored the high rate of physician attendance. Most of them (31) emphasized a low participation rate for several professional categories, especially anaesthetists in surgical wards and paramedical professionals. Another topic related to improvement of case selection with 14 comments. In particular, ten participants suggested reviewing more relevant cases. Concerning the organization of MMCs, 12 comments highlighted the lack of time available for healthcare professionals. Four professionals described an open and friendly environment, whereas eight described a blameful environment with conflicts and subjects that could not be debated.

Finally, a lack of a structured analysis of cases was reported. According to the respondents, the follow-up of initiatives should be improved as well as information provided outside the committee. A lack of involvement from the hospital administration was also pointed out.

## Discussion

Participants mostly agreed that MMCs were beneficial in terms of healthcare quality and safety improvement. MMCs were perceived as beneficial for teamwork and the functioning of the unit. The improvement of practices and organization seemed to be the main objective perceived by the participants. Moreover, an educational role, for initial and continuing education, was perceived by most participants, particularly by the senior physicians. Most of participants were satisfied with MMCs and experienced a friendly and non-blaming environment.

The search for failures and the discussion of errors are well-documented opportunities for improvement of safety and education [2, 14]. The present results suggest that the analysis of these failures was perceived as determinant in improving patient safety. However, without a thorough analysis, this discussion seems to result in a blaming environment that is incompatible with constructive discussion [6, 15–18]. Indeed, personal failures are often spontaneously discussed first. To overcome personal accusation and to develop a system-wide approach, a thorough analysis should be carried out using a systematic analysis method such as the protocol of the Association of Litigation and Risk Management (ALARM) [19]. However, due to the low number of MMCs that used a systematic method of analysis, it was not possible to study its impact. These methods are known and described, but it must be recognized that they are not commonly used [20]. The time constraints described by several professionals could be a reason for the underuse of a time-consuming method of analysis.

Moreover, the number and the type of cases discussed varied and affected the accuracy of analyses. Some authors suggest reviewing all deaths and complications to avoid missing opportunities to identify failures [21–23]. Others suggest performing a more thorough analysis based on a prior selection of relevant cases [4, 11, 14]. As the opportunity to spend more time on cases was positively perceived in this study, we would favor this method. According to numerous authors, the choice of cases should be based on errors or system safety issues [2, 16, 24].

The characteristics related to a formalized framework were associated with the perception of MMCs being more effective. This result is consistent with the literature and particularly with the impact of standardized presentations on participant satisfaction and on the production of improvement initiatives [10, 11]. This type of formalized framework could be valuable in providing more time for a system-oriented discussion [16].

The participants suggested including other professionals such as paramedical staff in the MMCs. Considering that incidents could involve all the healthcare professionals in a medical unit, this would allow a more accurate and system-oriented analysis [4, 6, 17, 25, 26] and increase the acceptance of improvement initiatives [16, 26]. Furthermore, paramedical professionals take an interest in safety management [27]. Such a team-based improvement meeting is also a way to establish a culture of safety within the department [16, 26].

We note that residents perceived fewer benefits than the other professionals. Our results do not suggest that they perceived MMCs as more blaming. Given that they change units every 6 months, they cannot perceive long-term improvement. Moreover, they are undoubtedly more focused on their personal training and therefore less motivated by team improvement.

A higher effectiveness index was associated with a greater perception of the effectiveness of MMCs in terms of patient safety. This suggests that an index based on the implementation of improvement initiatives could be a valuable way to assess the effectiveness of MMCs, although this hypothesis deserves further investigation based on clinical outcomes. The type of improvement initiatives and their follow-up are also relevant [28]. Moreover, the results of this study are consistent with those obtained previously concerning the characteristics associated with more effective MMCs.(P François, et al., submitted manuscript).

This study had several limitations. First, we assessed MMCs using the participants’ subjective perceptions. However, these perceptions are associated with the effectiveness index, a more objective measure of effectiveness. Second, we cannot exclude a selection bias and respondents could be particularly motivated or, on the other hand, more critical. Finally, this study was conducted in only three French hospitals. The development of MMCs is rather recent in France and they sometimes take place within specific settings. Consequently, the conclusions should be considered with regard to these specificities.

## Conclusions

In conclusion, this study showed the participants’ interest in MMCs. A thorough analysis of failures stands out as the main beneficial characteristic. The suggestions for improving MMCs concern a more structured framework and the attendance by a greater variety of professional categories in order to guide MMCs toward a system-wide approach. Furthermore, the improvement initiatives were associated with MMCs perceived as being more effective.
